# Transcriptomic timeseries links hepatic gene expression to an early and self-limited systemic response to enteric infection

**DOI:** 10.64898/2026.04.07.717127

**Published:** 2026-07-27

**Authors:** Yuko Hasegawa, Akina Osaki, Masataka Suzuki, Ting Yan, Ian W. Campbell, Matthew K. Waldor

## Abstract

The liver receives microbe and host signals from the intestine via the portal vein, thereby connecting the gut to systemic physiology. Homeostatic control of the timing of systemic responses is critical to prevent the expansion and dissemination of gut microbes and to mitigate untoward effects from prolonged systemic inflammation, however these mechanisms remain enigmatic. Here, to determine the role of the liver in coordinating systemic immune responses to enteric infection, matched measurements of global gene expression profiles were collected from the murine liver and intestinal epithelium throughout the course of enteric infection and clearance of *Citrobacter rodentium* , a mouse model of infectious colitis. These data revealed metabolic suppression in the liver during the peak of infection and a long-lived immune signaling pattern in the colon associated with CD4 and CD8 T cell infiltration that persisted beyond the clearance of infection. Furthermore, an early inflammatory signal was detected in the liver that resolved before the peak of disease and pathogen colonization. This self-limited, early signal depended on the pathogen’s virulence program and correlated with the timing of a corresponding systemic response, including circulating TNF-α and IL-6, key mediators of acute-phase proteins. These results uncover the temporal pattern of hepatic changes in response to the course of intestinal infection and provide correlative evidence that an early pulse of gene expression in the liver coordinates and limits the duration of the systemic acute-phase protein response.

**Author summary:**

Localized infections can trigger systemic inflammatory responses that help limit infection. However, prolonged systemic inflammation risks tissue damage and disease. Thus, the timing of systemic immune reactions is critical to the balance between immunity and damage. Yet, knowledge of the pathways that link gut-localized infections to systemic immune tone is limited. Since the liver is anatomically central to the connection between the gut and circulatory systems, we hypothesized that monitoring the kinetics of liver gene expression throughout the natural course of an intestinal bacterial infection would provide a valuable resource and yield insight into the control of systemic immune tone. Critically, we identified a burst of liver cytokine signaling that occurred and resolved before peak pathogen burden and disease in the colon and predicted the circulating inflammatory response. We propose that this early and self-limited signal from the liver coordinates the timing of the systemic response, ensuring it occurs early enough to promote immunity and resolve before causing tissue damage.

## Full Text Availability

The license terms selected by the author(s) for this preprint version do not permit archiving in PMC. The full text is available from the preprint server.

